# Optical, electrochemical and photocatalytic properties of cobalt doped CsPbCl_3_ nanostructures: a one-pot synthesis approach

**DOI:** 10.1038/s41598-021-95088-2

**Published:** 2021-08-13

**Authors:** Aadil Ahmad Bhat, Shakeel Ahmad Khandy, Ishtihadah Islam, Radha Tomar

**Affiliations:** 1 School of Studies in Chemistry, Jiwaji University, Gwalior, M.P 474011 India; 2 Department of Physics, National Taiwan University, Taipei, 10617 Taiwan; 3 Department of Physics, Jamia Milia Islamia, New Delhi, 110025 India

**Keywords:** Optical materials and structures, Materials for energy and catalysis, Materials for optics, Inorganic chemistry, Materials chemistry

## Abstract

The present manuscript aims at the synthesis of cesium based halide perovskite nanostructures and the effect of cobalt doping on the structural, optical, lumnisent, charge storage and photocatalytic properties. In a very first attempt, we report the solvothermal synthesis of Co doped CsPbCl_3_ nanostructures under subcritical conditions. The structural features were demonstrated by X-ray diffraction (XRD) Surface morphology determined cubic shape of the synthesized particles. Doping is an excellent way to modify the properties of host material in particular to the electronic structure or optical properties. Incorporation of Co^2+^ ions in the perovskite structure tunes the optical properties of the nanostructures making this perovskite a visible light active material (Eg = 1.6 eV). This modification in the optical behaviour is the result of size effect, the crystallite size of the doped nanostructures increases with cobalt doping concentration. Photolumniscance (PL) study indicated that CsPbCl_3_ exhibited Blue emission. Thermogravametric analysis (TGA) revealed that the nanostructures are quite stable at elavated temperatures. The electrochemical performance depicts the pseudocapacative nature of the synthesized nanostructures and can used for charge storage devices. The charge storage capability showed direct proportionality with cobalt ion concentration. And Finally the photocatalytic performance of synthesized material shows superior catalytic ability degrading 90% of methylene blue (MB) dye in 180 min under visible light conditions.

## Introduction

**Figure 1:**
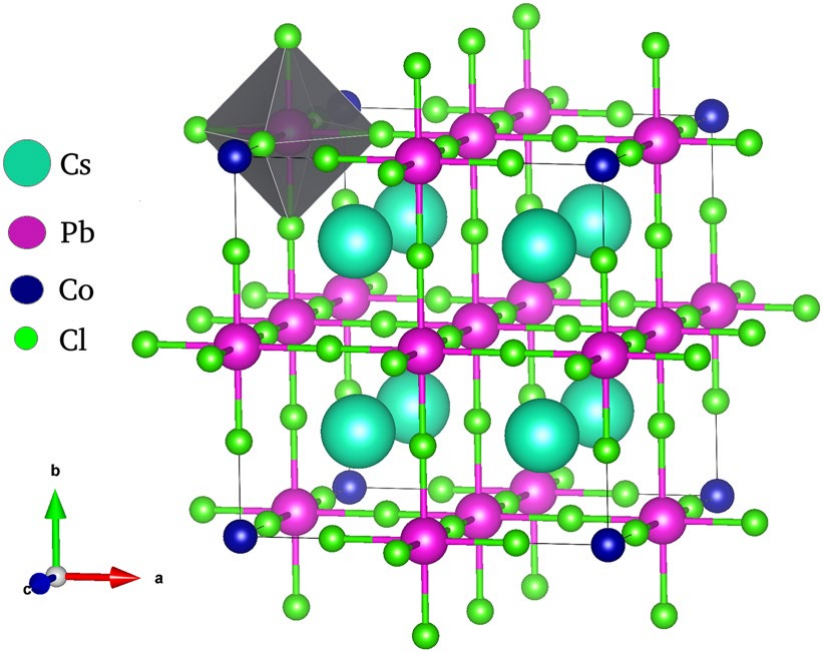
Possible crystal structure of 15% Co- doped tetragonal CsPbCl_3_ halide perovskite.

Perovskite nanostructures have revolutionized the power gneration capacity of Solar plants because of theirvibrant functionalities insolar cell and engenering techonolgies. Metal Halide Perovskites (MHP) are initially used as sensiting materials for solar cells which have capablity of retaining high charge transport properties^1,2^. The caping agents used for the synthesis of metal halide perovskite control the growth of the nanostructures. It is possible to tune the size and shape of nanomaterial so that the synthesis of bulk nanocrystals as well as nanostructures like nanwires, nanosheets and quantum dotsis achieved^3,4^. A little bit overview of the nanocrystal size can be tuned not only during the synthesis but also by post synthesis transformation via ion exchange reaction mechanism^5,6^. However, Cesium based halide perovskite are considered to be the most reliable materials for perovskite solar cells (PSC), light emitting diodes (LED’s) and photo detectors due to their higher thermal stability in comparison to the hybrid perovskites^7,8^. The performance of displaying tuneable band gap and large diffusion length along with large life time specify their valuable characteristics. These striking properties are mostly depend upon the size and morphology of the nanomaterial. Aboundant attempts had been put forword to improve the efficiency and stability of Metal halide perovskite by doping various transition, post transition or lanthanoids elements for energy harvesting device applications^9,10^. Doping is an excellent way to modify the inhertant optical, magnetic and electronic properties of the host material^11,12^. The photolumniscence quantum yield reaches upto 90% for CPbBr_3_ and CsPbI_3_, which display green and red emission, respectively^13,14^. CsPbCl_3_ has nearly 100% PL quantum yield on incorporating Cd ions in its structure^15^. Various transition metal and lanthanoids were reasonably doped in CsPbCl_3_ to reduce the non radiative defectsand toxitityof nanomaterial^16,17^. From the last several years various number of metals includeing Mn^2+^, Zn^2+^, Ti^2+^ and also lanthanoids elements such as Ce^3+^ and Yb^3+^ have been sucessfully incorporated in the nanostructured CsPbCl_3_ perovskite^18,19^. Doping of Mn transition metal in CsPbCl_3_ emerges a new emission spike in photolumniscence (PL). However, the PL activity of the nanocrystal is enhanced by increasing the concentration of transition element in the host material. At the same time, higher concentration of [Mn, Fe and Co] overpowers the PL and electronic properties of CsPbCl_3_ materia^20,21^. However uniform doping in one dimentional perovskite is a difficult task because it needs homoginity in both cross section and in longtudinal/axial growth direction. One dimensioal nanostructures, nanowires, nanorods and nanotubes have found immense properties in the optoelectronic devices and applications^22,23^. MHP are mainly rely on the use of divalent metal lead. However, there are increasing threat regarding the poisonous nature of Pb which is concerned to health and the environment. Restriction of hazardous substances directive severely limits the use of Pb in consumer electronics. There have been a number of attempts to explore and replace lead with less toxic elements, having an analogous electronic band structure to that of Mn Fe, and Co. Moreover, Cobalt seems an appropriate choice because it has an electronic configuration of 3d^7^ and 4S^2^. Beyond being lead-free, Cobalt halide perovskites shows a number of properties which make them attractive for use in photovoltaic including narrower band gaps than their lead analogues, low exciton binding energies, and long carrier diffusion lengths. In this regard, CsPbCl_3_ can be synthesised by different methods e.g. Hot injection method, However, Cao Z. et al. report the effect of Co^2+^ in the Mn doped CsPbCl_3_ perovskite nanocrystals through hydrothermal approach^24^. Although,CsPbCl_3_ can be synthesised by different methods e.g. Hot injection method, but we prefer the hydrothermal synthesis because it controls the growth and crystallinity of the material. The typical crystal structure of CsPbCl_3_ nanomaterial is shown in Fig. 1.

To the best of our knowledge no report on the synthesized Cobalt doped CsPbCl_3_ nanostructures by Solvothermal method is available in the literature. The present manuscript hence, aims at the synthesis of CsPbCl_3_ nanostructures and the possible effect of Cobalt doping on CsPbCl_3_ its properties. The structural, optical, photoluminescence and electrochemical properties are demonstrated in the below section of this manuscript.

## Experimental section

### Materials and synthesis

All the chemicals and reagents are of analytical grade (AR), purchased from CDH Chemicals and were used without further purification. To synthesize Cesium lead based halide perovskite, 0.5 M of CsCl_2_ and 0.5 M PbCl_2_ were dissolved in 5 ml of Dimethyl sulphoxide (DMSO). 10 μL of HCl were added to the reaction mixture to avoid precipitate formation. The mixture was in sonication till a clear solution was formed. To this solution 50 ml boiling toluene was added drop wise resulting in the formation of slurry mixture. This slurry was in transferred into a stainless steel autoclave (100 ml) capacity. The autoclave was subjected to a temperature of 130 °C for 8 h after the reaction conditions were full filled, the autoclave was cooled to room temperature on its own. The settled material at bottom of the autoclave is collected and washed with toluene. (3 times). The filtered pink material was in dried at 140 °C for 30 min. The powered thus obtained was captioned as sample CsPbCl_3_. Three more samples were prepared in which 5%, 10% and 15% Cobalt doping was introduced in place of lead and the samples were captioned as 5% Co:CsPbCl_3_, 10%, 15% respectively.

All the samples synthesized were vacuum annealed at 250 °C for 30 min to remove the unreacted precursors and byproducts.

### Fabrication of glassy carbon electrode (GCE)

The electrochemical properties of pristine and Cobalt doped CsPbCl_3_ nanostructures were carried out by cyclic voltammetry. Prior to use, the Glassy Carbon electrode was well polished with 0.5 μm alumina powder and washed with distilled water several times followed by drying at room temperature. Then about 0.1 g of synthesized material was dispersed in 2 ml of ethanol and add 2 drops of binder (NMP) to it. In order to achieve uniform dispersion of synthesized material, the whole mixture undergoes sonication for about 15 min. Then about 10 μL of suspension is drop casted on the GCL at dried it overnight. Then the dried GCE is used for electrochemical analysis.

### Characterization

In order to figure out structural, optical, compositional, morphology, luminescent and capacitance properties have been carried out by XRD (Rigaku Miniflex-600), UV–Visible spectroscopy (UV-2450 Shimadzu), SEM (Philips, Model-Quanta 200 FEG) and Spectrofluorophotometry (RF-6000 Shimadzu), Auto lab type lll potentiostat, respectively.

## Results and discussion

### XRD analysis

**Figure 2:**
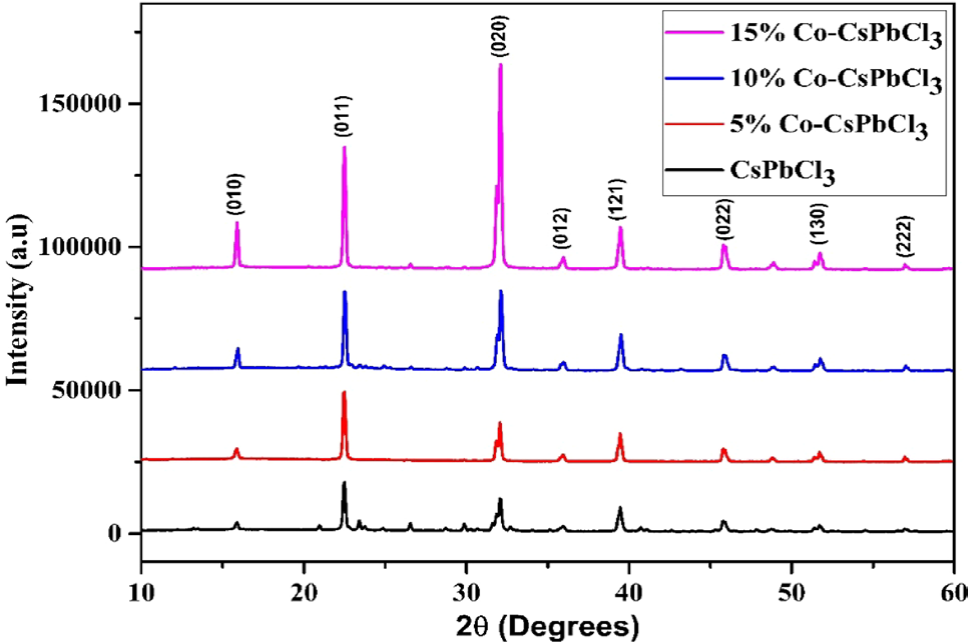
XRD graph of the Pristine and Co doped CsPbCl_3_ nanostructures.

Figure 2 depicts all the diffraction peaks of the CsPbCl_3_ nanostructures. The diffraction peaks at (15.88), (22.45), (32.10), (35.91), (39.48), (45.86), (51.70) correspond to their miller indices (010), (011), (020), (012), (121), (022), (130), (222), respectively, which resemble with the tetragonal phase of CsPbCl_3_ having space symmetry of P4mm [JCPDS No. 18 # 0366].

Both the pristine and Co:CsPbCl_3_ samples show the tetragonal phase of the crystal lattice. From the XRD pattern it is concluded that the decrease in concentration of dopant increases the peak intensity as well as crystallinity of the prepared samples. No other peaks have been found in the XRD graph which shows that the samples are highly pure. Upon increase in concentration of Co-dopant in the host lattice, the crystallite size of the material increases and the lattice strain decreases vice versa. The crystallite size of the nanostructures was calculated by Scherer equation

1 $$ D = {\raise0.7ex\hbox{${0.9*\lambda }$} \!\mathord{\left/ {\vphantom {{0.9*\lambda } {\beta {\text{Cos}} \theta }}}\right.\kern-\nulldelimiterspace} \!\lower0.7ex\hbox{${\beta {\text{Cos}} \theta }$}} $$

The lattice constant of the tetragonal crystal can be estimated from the given expression.

2 $$ \frac{1}{{d^{2} }} = \frac{{h^{2} + k^{2} }}{{a^{2} }} + \frac{{l^{2} }}{{c^{2} }} $$

where d is the interplaner spaceing, a and c are lattice constants and h, k,l are the miller indices. The lattice constant of all the samples were calculated by taking miller indicies 011 and 020, their 2θ value is approximately at 22 and 32 respeactively. The lattice constant, Crystallite size and strain of the pristine CsPbCl_3_ and Co doped CsPbCl_3_ crystal are shown in Table 1.

**Table1:** Represents the lattice parameter, crystallite size, strain of Pristine and Co doped CsPbCl_3_ nanostructures.

S. no.	Sample name	Lattice constant (Å)		Crystallite size (nm)	Lattice strain
		a = b	c		
1	CsPbCl_3_	5.57	5.59	50.08	0.0037
2	5% Co-CsPbCl_3_	5.55	5.59	51.98	0.0036
3	10% Co-CsPbCl_3_	5.55	5.61	52.9	0.0035
4	15% Co-CsPbCl_3_	5.55	5.61	53.6	0.0030

### FTIR analysis

**Figure 3:**
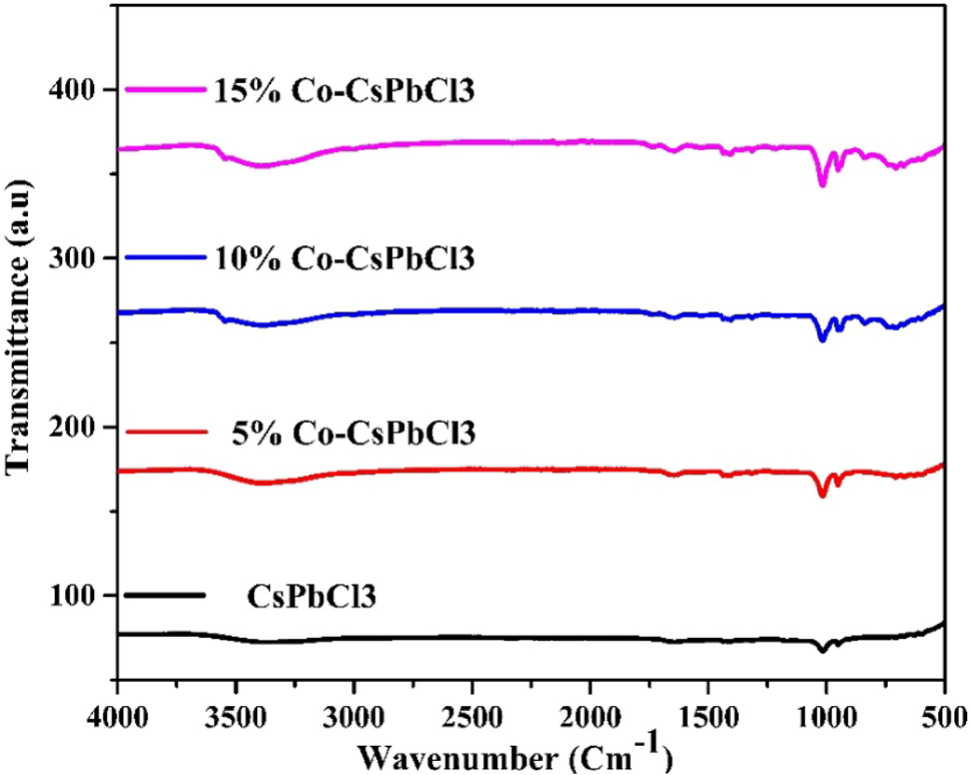
FTIR spectra of Pristine and Co:CsPbCl_3_ nanostructures.

Detection of functional groups can be estimated by Fourier transformation infrared spectroscopy. The FTIR spectra of pristine CsPbCl_3_ and Co:CsPbCl_3_ nanostructures are shown in Fig. 3. The samples show absorption peak in the range of 3386 Cm^−1^ corresponding to the OH group. The peak at 1654 Cm^−1^ is ascribed due to the presence of C=O. While the peak at 1019 Cm^−1^ is due to the presence of CH_2_ bond, which comes from the solvent. The transmittance peak at 947 Cm^−1^ and 725 Cm^−1^ is ascribed due to bending and rocking vibration of CH_2_^25^. Thus, FTIR spectra confirms the formation of pristine and Co doped CsPbCl_3_ nanostructures.

### Thermo-gravimetric analysis (TGA)

**Figure 4:**
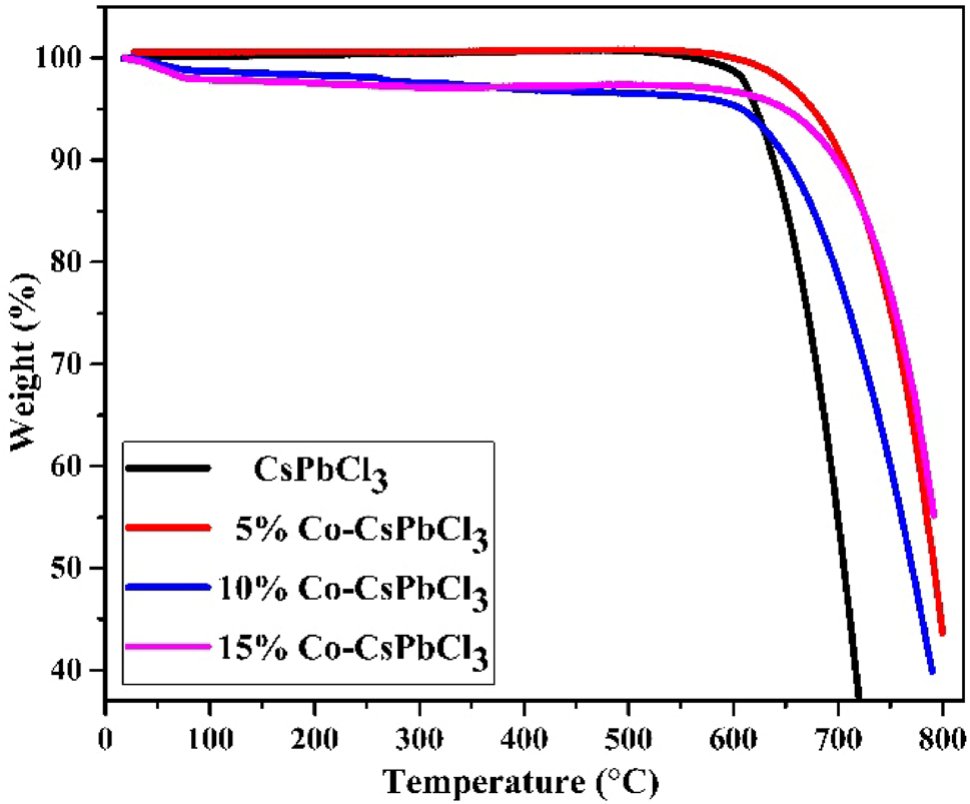
The TG plot of CsPbCl_3_ and Co:CsPbCl_3_ nanomaterial.

In order to investigate the thermal stability of Synthesized CsPbCl_3_ and Co doped CsPbCl_3_ nanostructures, thermo gravimetric analysis has been carried out^26^. The Cesium and its doped halide were heated from room temperature to 800 °C with an increment of 10 °C/min in presence of nitrogen atmosphere. Figure 4 shows TG plots of all the four samples. It is evident from the TG plot that none of the samples show weight loss upto 600 °C, which means that these materials are highly stable at elevated temperatures. However, there is sharp single –stage degradation of CsPbCl_3_ and Co:CsPbCl_3_ without any lower temperature features. In nutshell, the TG analysis predicts the higher thermal stability of all inorganic Cs based halide perovskite samples. Therefore these materials are more favorable for optoelectronic device applications.

### Morphological analysis

**Figure 5:**
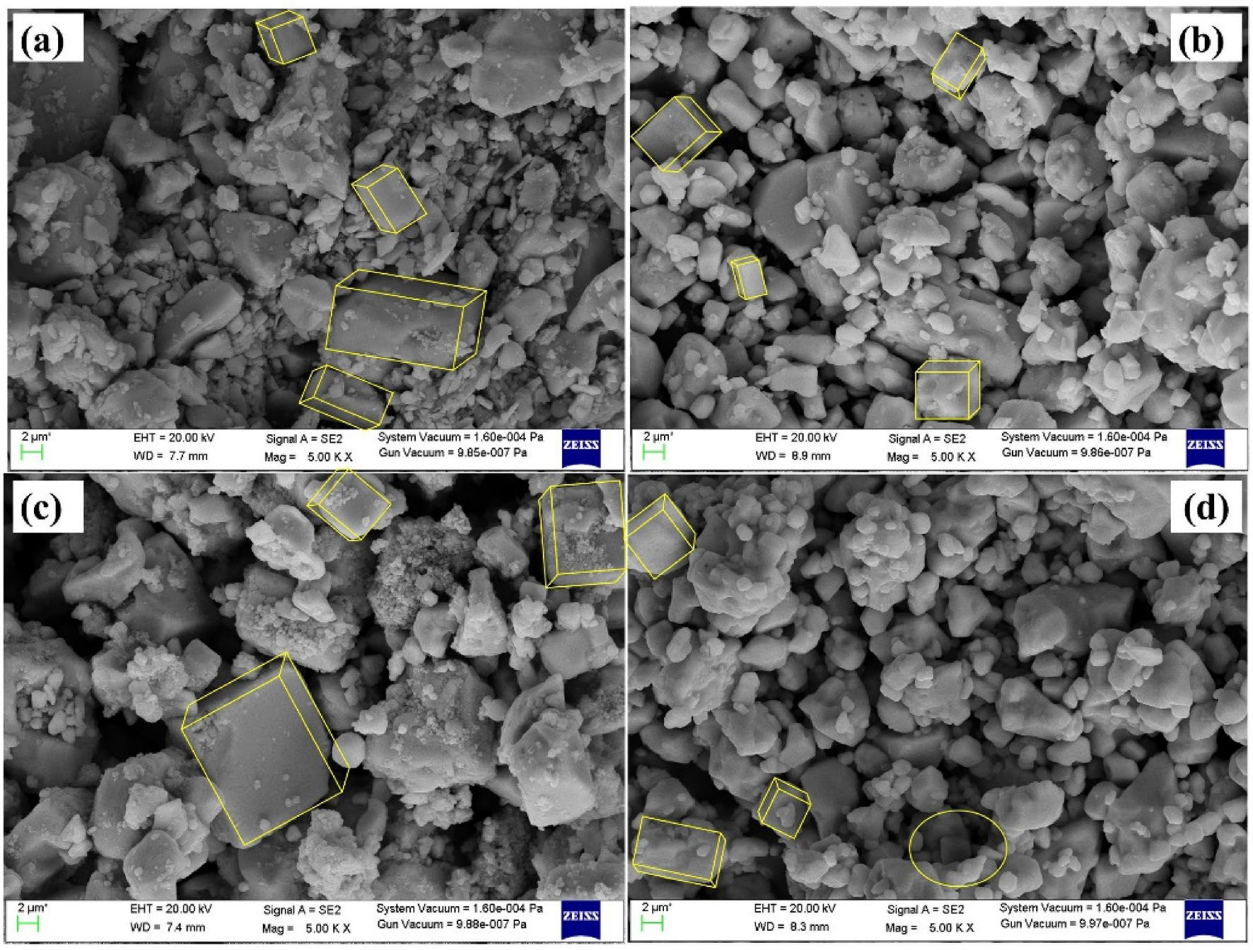
Micrograph of CsPbCl_3_ and Co doped CsPbCl_3_ nanostructures.

The surface morphology of a typical synthesized material CsPbCl_3_ and Co doped CsPbCl_3_ was illustrated by FESEM. The SEM micrographs of Pristine and [5%, 10%, 15%] Co doped CsPbCl_3_ are revealed in Fig. 5. From these micrographs it is evident that all the four samples exhibit cubic morphology/configuration. However, on increasing the concentration of Cobalt content up to 15% in the host material, the particle size is reduced. Due to this reduction these synthesized material are used for surface-active agents.

**Figure 6:**
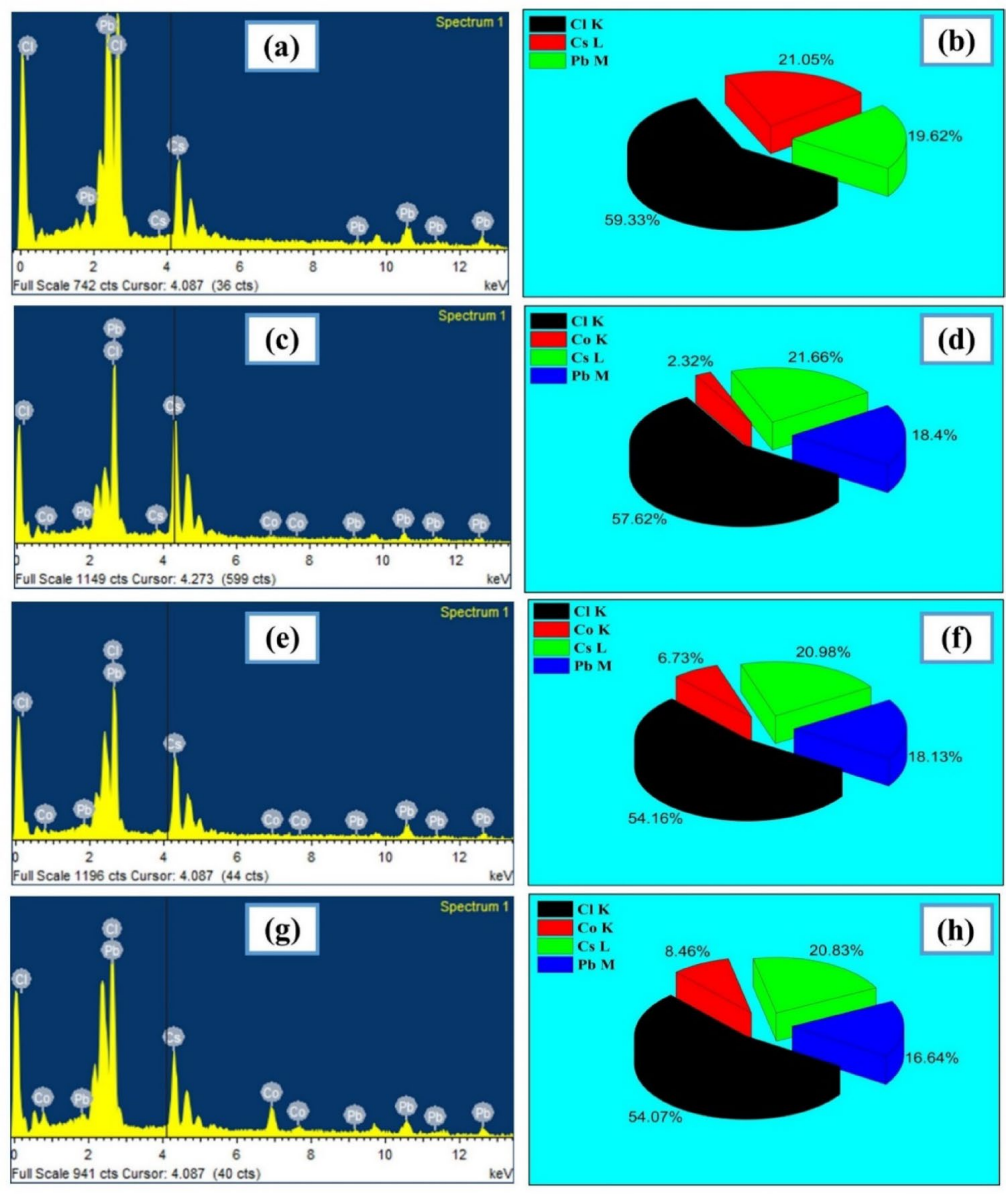
The EDS and Atomic Composition of CsPbCl3 and Co:CsPbCl_3_.

The EDS as well as atomic percentage of CsPbCl_3_ and Co:CsPbCl_3_ are shown in Fig. 6. From graphical interpretation of EDS clearly hints that only desired elements are found in the given samples, which means that there is no trace of impurity present in these nanostructures. The atomic percentage of Cs, Pb and Cl are approximately in the ratio of 1:1:3. While on increasing the concentration of Cobalt in the host lattice, there is a replacement of Pb rather than Cs. Because both Cobalt and Pb are of comparable size and maintain the charge neutrality of the host lattice.

## Optical analysis

**Figure 7:**
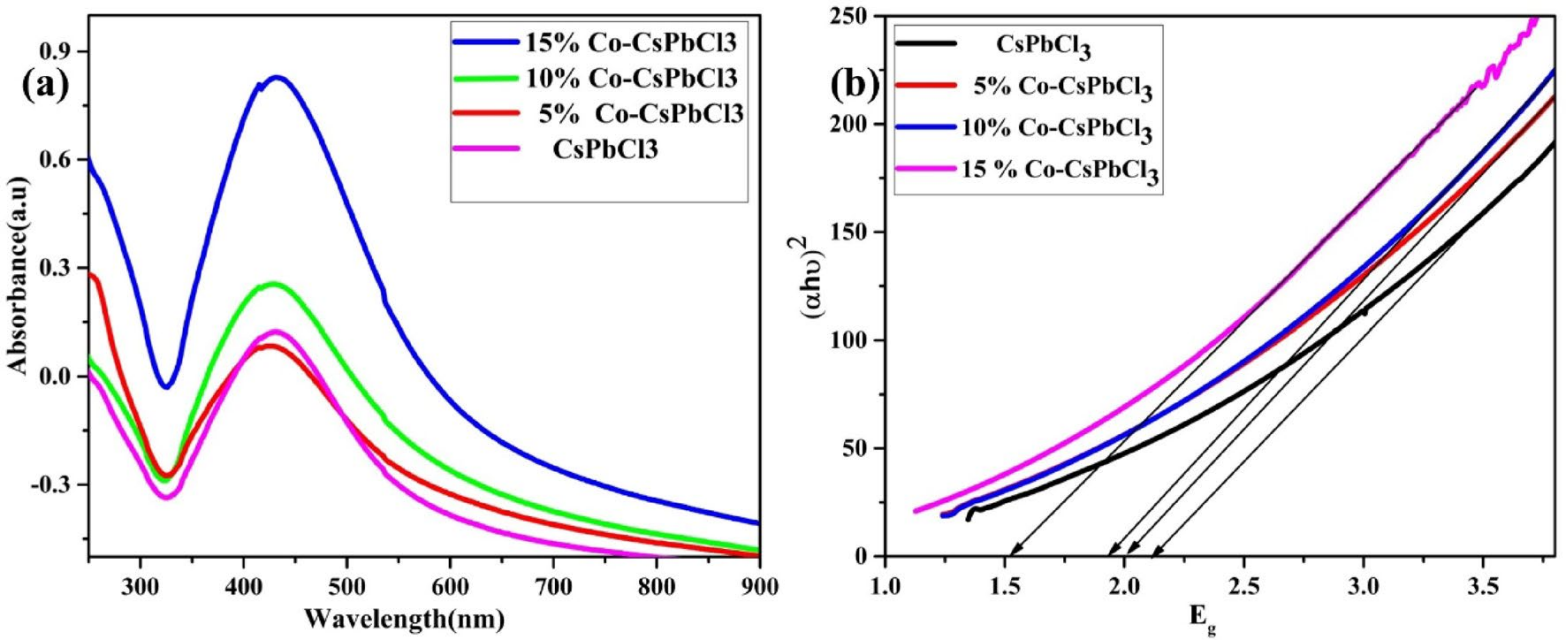
(**a**) Optical absorption onset and Tauc plot of CsPbCl_3_ and (**b**) Co:CsPbCl_3_ respectively.

In order to analyze the characterized optical absorption of synthesized nano-materials CsPbCl_3_ and Co doped CsPbCl_3_ by using the UV–Visible spectrophotometer. With the help of UV–Visible spectrophotometer we have plotted the optical absorption of all the four samples as labelled in Fig. 7a. The optical absorption of mentioned materials are active in the visible region. However, from the graphical representation it is evident that on increasing the concentration of dopant from 0–15% of cobalt ion in the host material there occurs a slight shift in the absorption peak to higher wavelength which is due to the fact of impurity defects. On the occasion, the absorbance intensity increases which is attributed due to increase in percentage of Cobalt. Furthermore, the band gap of material is tuned up to 1.5 eV upon 15% of cobalt doping which is estimated from the tauc’s plot (see Fig. 7b). Therefore, these tunable band gap materials can be used for photovoltaic solar cell applications.

**Figure 8:**
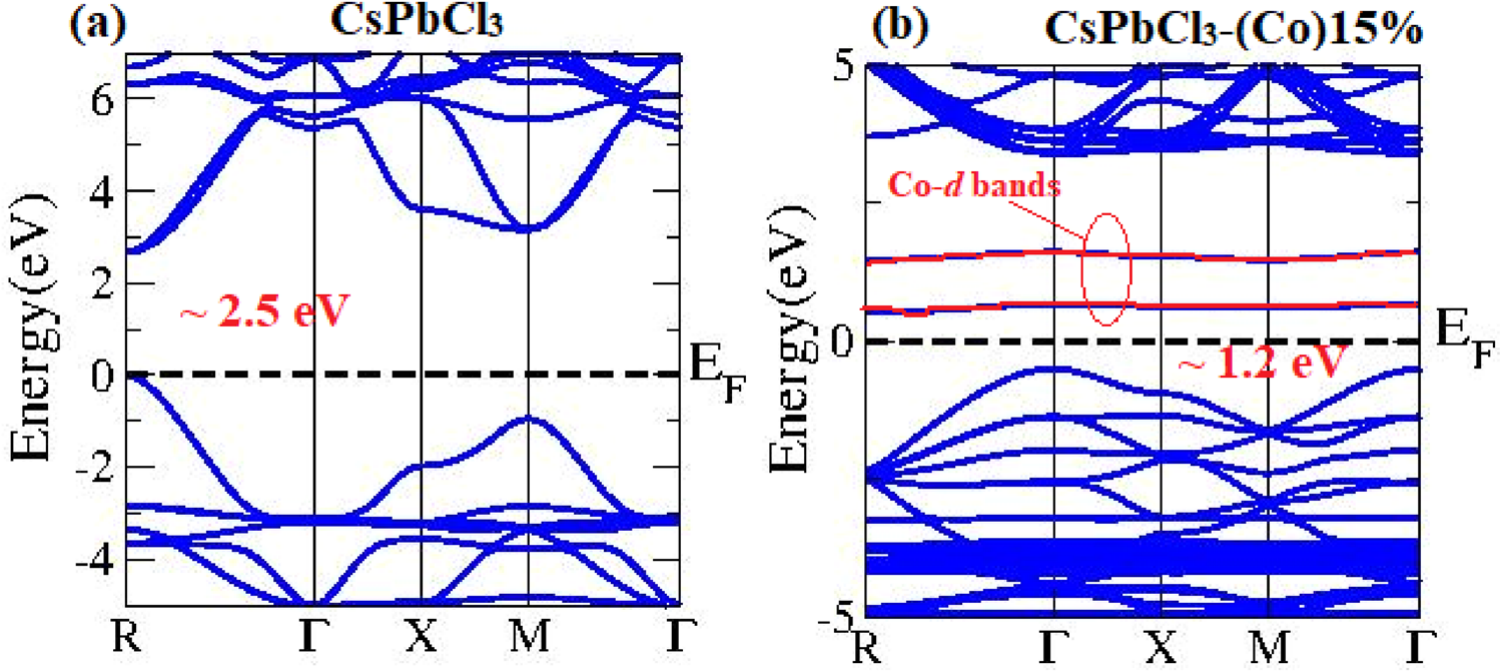
Electronic structure of (**a**) CsPbCl_3_ and (**b**) 15%-Co:CsPbCl_3_ simulated via DFT calculations.

Using DFT calculations, we simulated the pristine CsPbCl_3_ and 15% Co:CsPbCl_3_ to have a thorough understanding of the location of impurity states of Co in the host lattice. We make use of crystal parameters observed from XRD analysis of these samples and follow the modified Beckhe-Jhonsosn (mBJ) exchange correlation method for the electronic structure calculation in Wien2K Code^27–29^. DFT simulated band structures follow the experimental data sets with good accuracy and the supposed band gaps are ~ 2.5 eV in pristine (Fig. 8a) and ~ 1.2 eV in Co-doped CsPbCl_3_ samples (Fig. 8b). We can see the impurity Co-*d* states lying down the bottom of the conduction band, which hence act as the transition states during absorption process. This decrease in band gap originates from the Co^2+^atoms, which provide the partially filled d-orbitals for the electrons to hybridize near the Fermi level. Thus, we can say that the more the Co-dopants added in the host CsPbCl_3_ lattice, the lesser is the gap between valence band maxima and conduction band minima, provided the crystal structure remains intact and no structural phase transition occurs.

### Photo-luminescence properties

**Figure 9:**
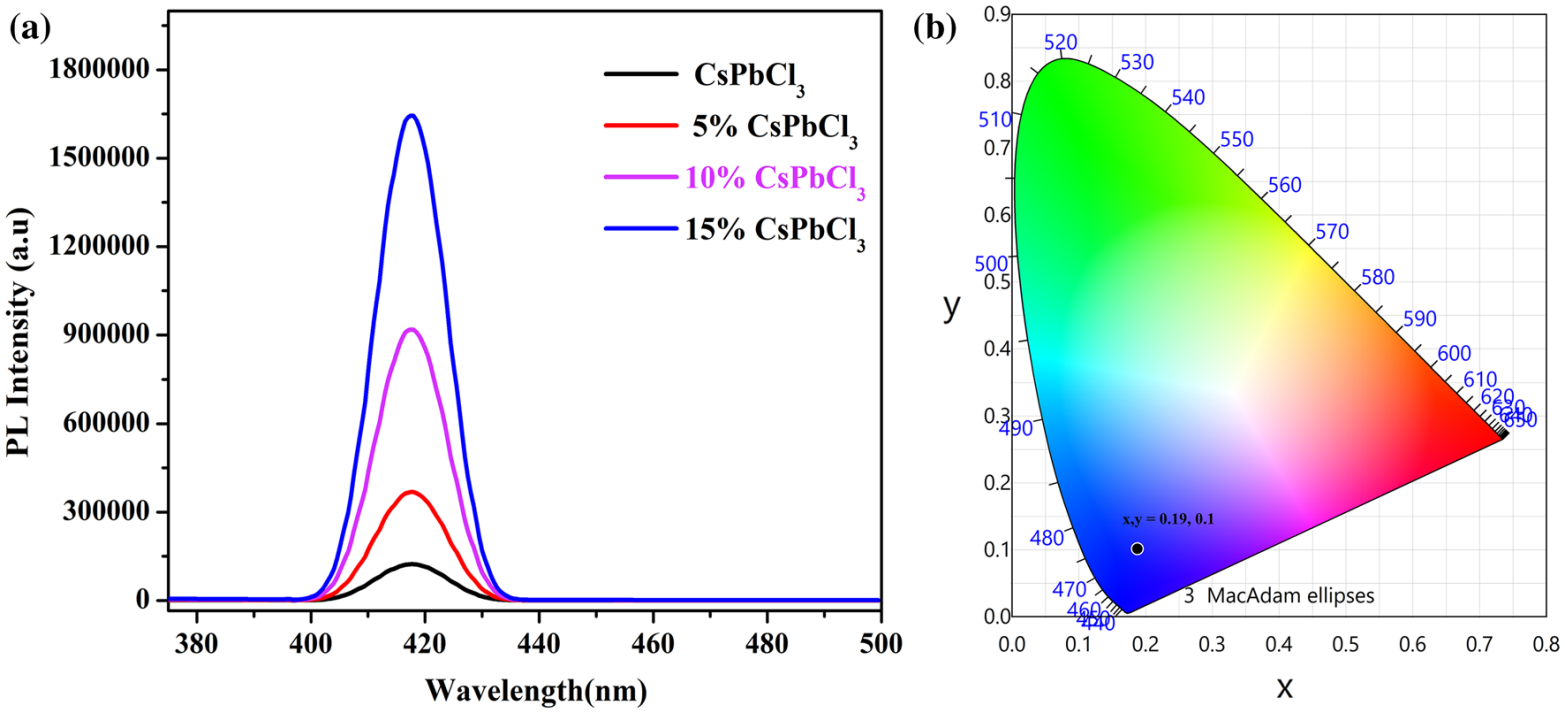
(**a**, **b**) The emission spectrum and CIE diagram of CsPbCl_3_ and Co:CsPbCl_3_, respectively.

In order to investigate the emission stability of synthesized CsPbCl_3_ and Co doped CsPbCl_3_ perovskite nanostructures, Spectrofluorophotometry (RF) was carried out. The emission spectra of all the four samples are shown in Fig. 9a. Their emission spectra was recorded at the excitation wavelength of 360 nm. It is clearly examined from the PL emission that the concentration of dopant atoms in the host lattice produces a slight blue shift in the Pl emission, which is due to the replacement of lead by Cobalt atoms. Due to this replacement, there occurs a lattice contraction in the host material^30–32^. Moreover, the CIE diagram of the material is shown in Fig. 9b. The synthesized material shows color coordinate at (x,y = 0.19, 0.1). From the above discussion it is pointed out that these materials may likely find applications in Blue emission LED’s.

## Electro-chemical analysis

**Figure 10:**
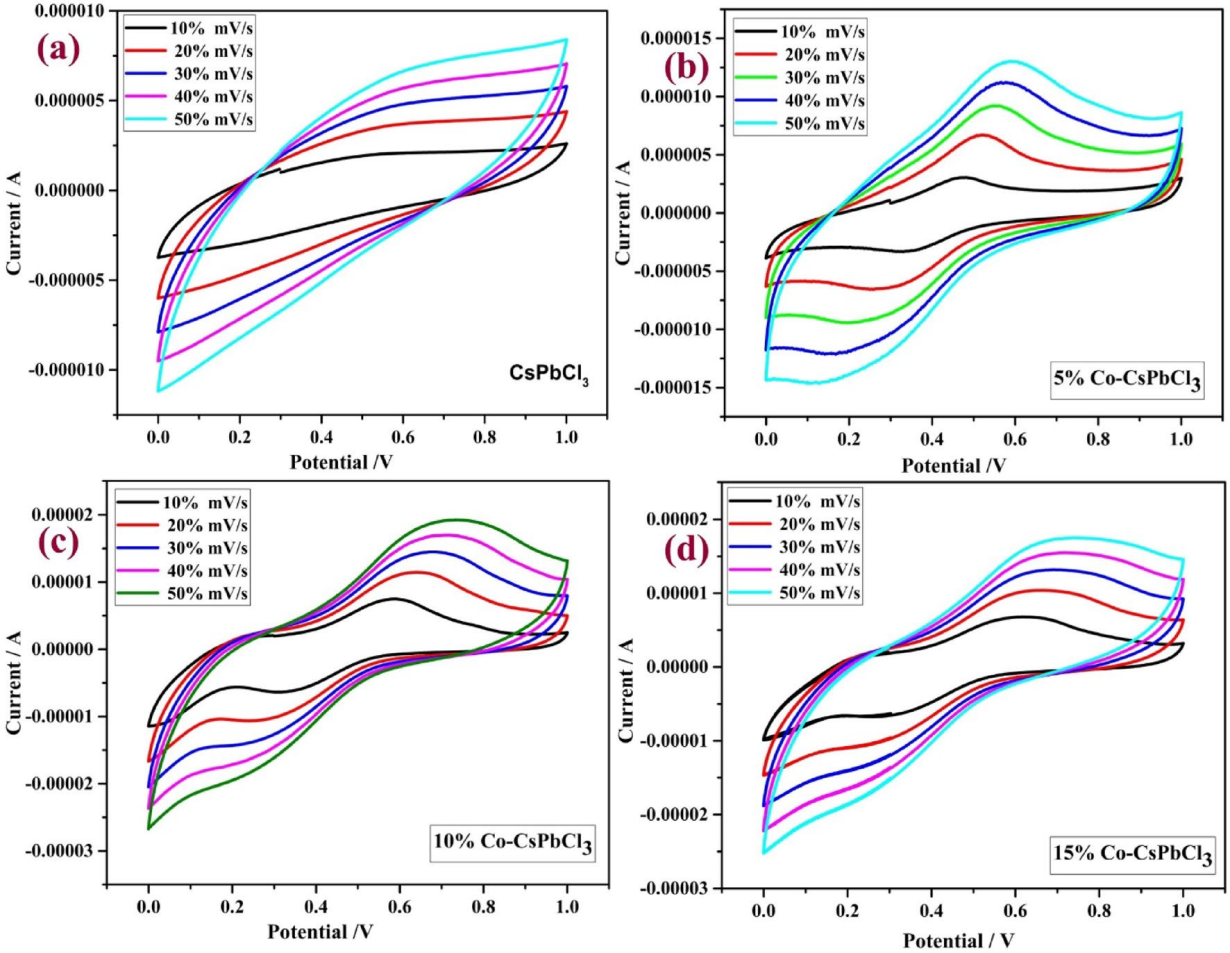
Represents the voltammogram of CsPbCl_3_ and Co:CsPbCl_3_.

In order to investigate the electrochemical performance of Cobalt doped CsPbCl_3_ nanostructures, cyclic voltammetry measurements were carried out at room temperature. The setup of CV consists of three-electrode system, Ag/AgCl electrode acts as reference, Pt wires behave as counter electrode and finally the GCE acts as working electrode. To check the electrochemical activity of synthesized material, 0.5 M H_2_SO_4_ is used as an electrolyte. A well oxidation and reduction peak was observed at the bare electrode. Figure 10 shows the voltammogram of all the four samples at different scan rates in between the potential range of 0 V to + 1 V.

From these voltammogram plots, it is evident that all the samples show pseudo capacitor behavior. The charge storage capability of the material can be calculated from the given expression.

3 $$ Cp = \frac{A}{MKV} $$

The calculated specific capacitance of all the samples are shown in Table 2.

**Table 2:** The specific capacitance at different Scan rates.

Sample	Specific capacitance (F/g)				
	10 mV/s	20 mV/s	30 mV/s	40 mV/s	50 mV/s
CsPbCl_3_	75.6	50	35.6	31	28.2
5% Co-CsPbCl_3_	88.2	70.8	55.93	52.5	50
10% Co-CsPbCl_3_	117.2	95.8	81.33	71.5	64
15% Co-CsPbCl_3_	154	122	101.3	87.5	77.6

**Figure 11:**
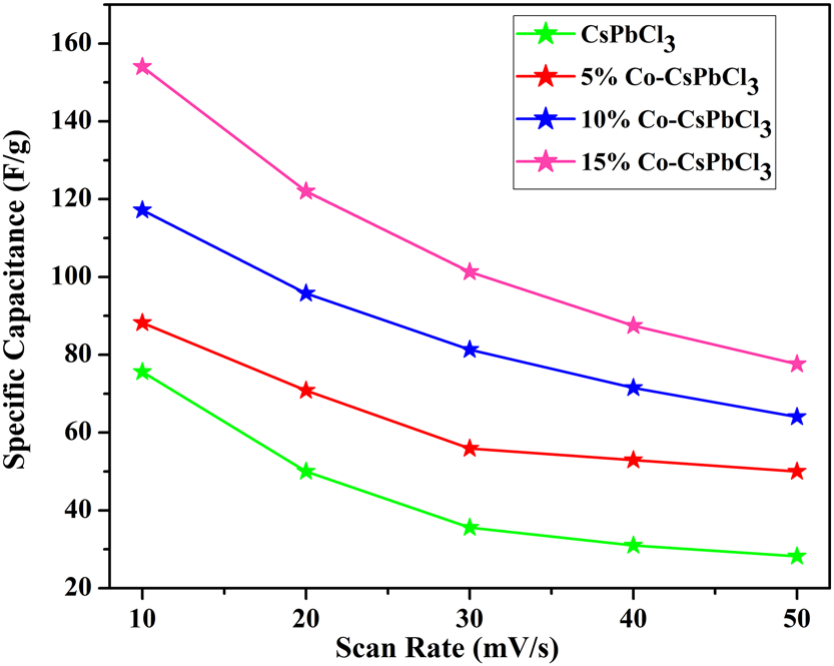
Specific capacitance of CsPbCl_3_ and Co:CsPbCl_3_.

From the Table 2, it is convenient that as the concentration of cobalt increases in the host material the charge storage capacity increases. While on increasing the scan rate, the specific capacitance decreases reason being the less contact between the electrode and electrolyte. Figure 11 shows the specific capacitance of all the nanostructured samples.

The value of current density increases as the scan rate increases while the shape of the voltammogram remains constant. The current density (*i*) of voltammgam can be estimated by the equation.

4 $$ i = (2.69*10^{5} .A.D^{1/2} .C.v^{3/2} ) $$

where v is the scan rate (Vs^−1^), A is the area of electrode (cm^2^), D is the diffusion cofficient and C is the concentration of electrode active species in the electrolyte (molL^−1^)^33^. A linear corelation of peak current and square root of scan rate shows the electrode reactionsas adiffusion controled process.

**Figure 12:**
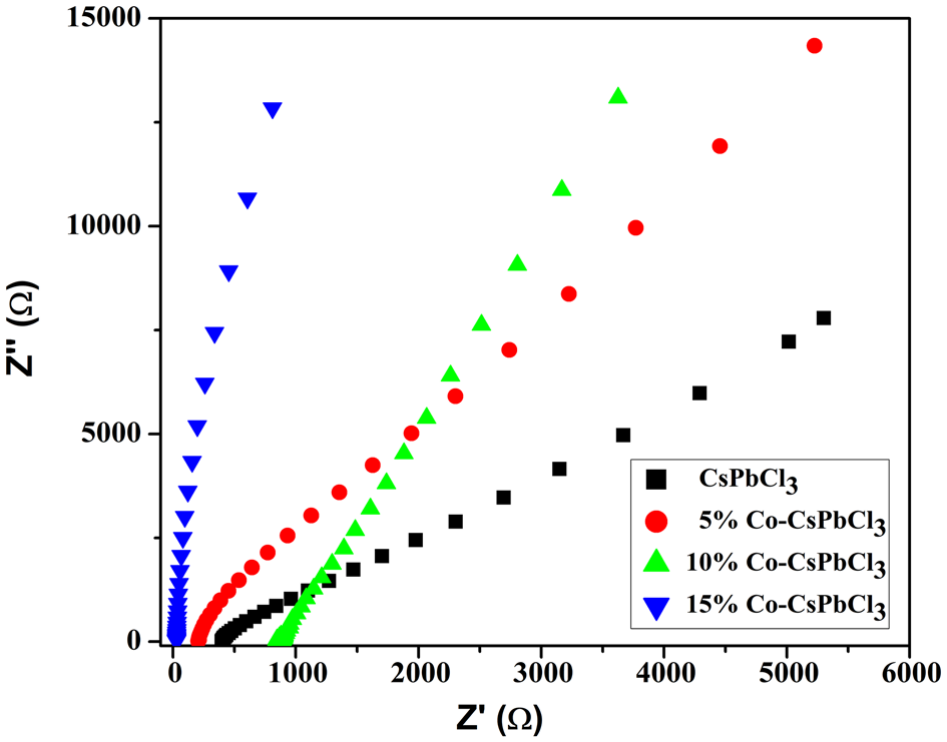
Nyquest plot of CsPbCl_3_ and Co:CsPbCl_3_.

The Nyquest plot of all the four samples are described in Fig. 12. It is evident from the plot that all the samples show slight linearity in the low frequency region. The linear part indicating the capacative behaviour of electrode system signifying the diffusion process^34^. The EIS data can be varified from table which is based on randle’s model system as shown in Table 3.

**Table 3:** Represent Rs and Rp of CsPbCl_3_ and Co:CsPbCl_3_.

S. no.	Sample name	Rs	Rp
1	CsPbCl_3_	833 Ω	166 kΩ
2	5% Co:CsPbCl_3_	204 Ω	14.2 kΩ
3	10% Co:CsPbCl_3_	17.6 Ω	16.2 kΩ
4	15% Co:CsPbCl_3_	-173 Ω	174 kΩ

The table consists of ohmic resistance (Rs), Polarisation resistance (Rp). As the concentration of dopant increses the ohmic resistance decreases, which favours the heigher percentage of doping.

## Photo catalytic activity

**Figure 13:**
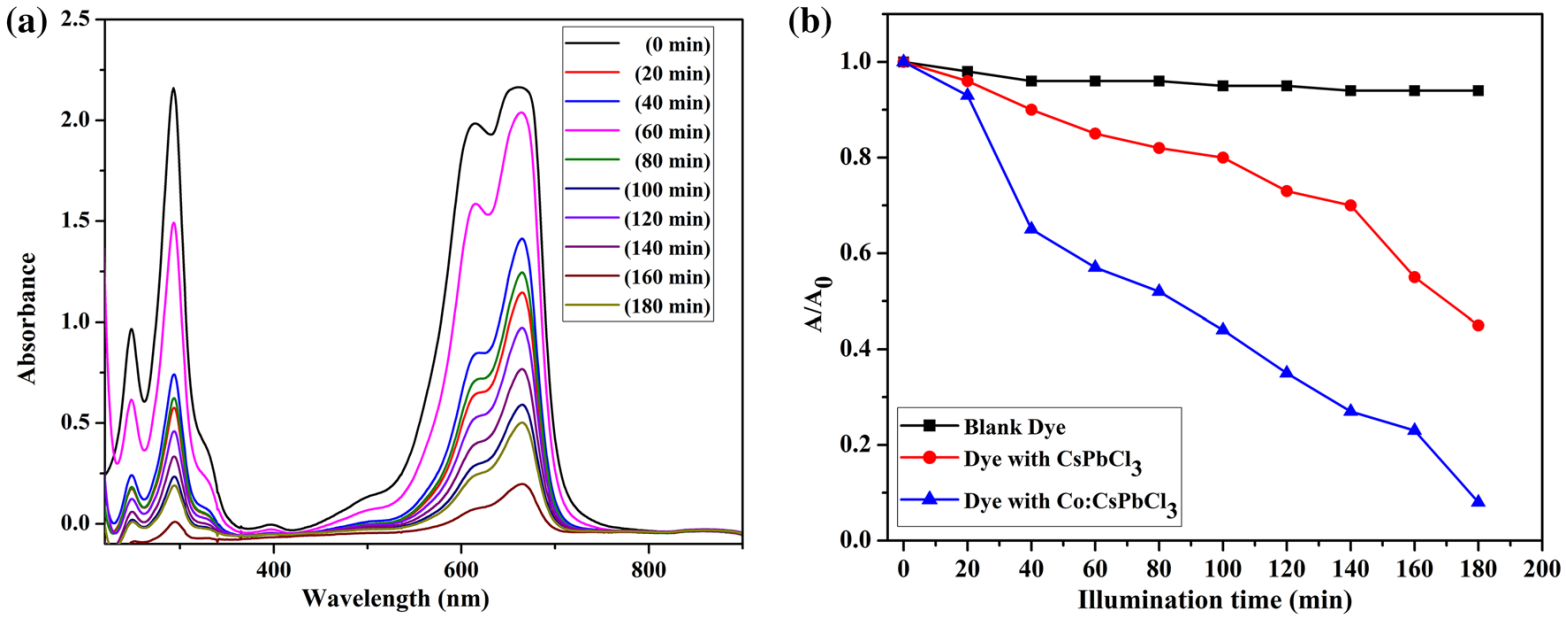
(**a**, **b**) The effect of UV–Visible spectra of MB and decrease in con. of MB with time respectively.

The photocatalytic activity of the nanoparticles was explored against MB dye and for this sample 10% Co:CsPbCl_3_ was tested because of its superior structural and morphological properties. The photocatalytic experiments were carried out in a self made photocatalytic reactor reported in other publications^35,36^. The photocatalytic activity of the nanostructures was demonstrated under visible light illumination. The activity was tested against 20 ppm MB and the catalyst loaded was 2 mg. The mixture containing the dye and the catalyst was stirred under dark. The solution stirred under dark was tested for absorbance and this observation was carried out after regular intervals until we get static absorbance. This behaviour revealed attainment of adsorption–desorption equilibrium. After this the catalyst treated dye solution was illuminated with visible light. After illumination, the absorbance was observed after regular time intervals using the UV–Visible spectrophotometer. The time dependent absorbance spectra are shown in Fig. 13(a). The spectra have a gradual falls with illumination time. The fall represents decrease in the concentration of the dye in the solution as per Lambert–Beer law.

It was found that more than 90% of the dye was degraded in 3 h of light illumination, which is a good activity. The synthesized catalyst is having quite good activity in comparison with the reported literature. Concentration curves for blank dye solution, undoped catalyst treated and doped catalyst treated dye solutions in presence of visible light are shown in Fig. 13(b). It is quite clear from the plot that sample 10% Co:CsPbCl_3_ has better activity than the undoped sample.

The MB degradation followed pseudo first order kinetics. The pseudo first order expression is shown below.

5 $$ \ln (C_{0} /C) = KC $$

where K is the pseudo first order rate constant. The rate constant (K) can be calculated from the slope, of the plot between the ln (C_0_/C) Vs time and its value calculated is 10.98 × 10^–3^.

**Figure 14:**
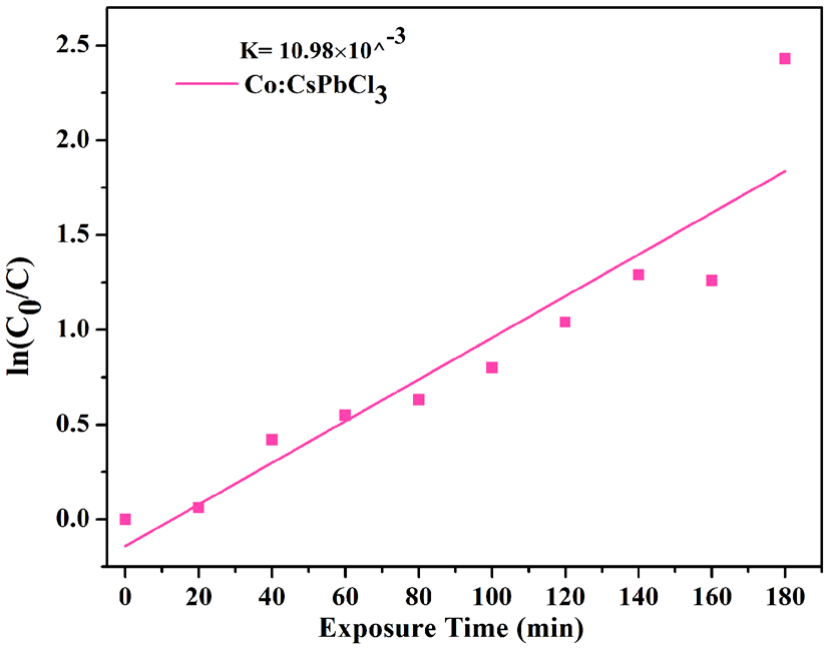
The slope of Co:CsPbCl_3_.

**Figure 15:**
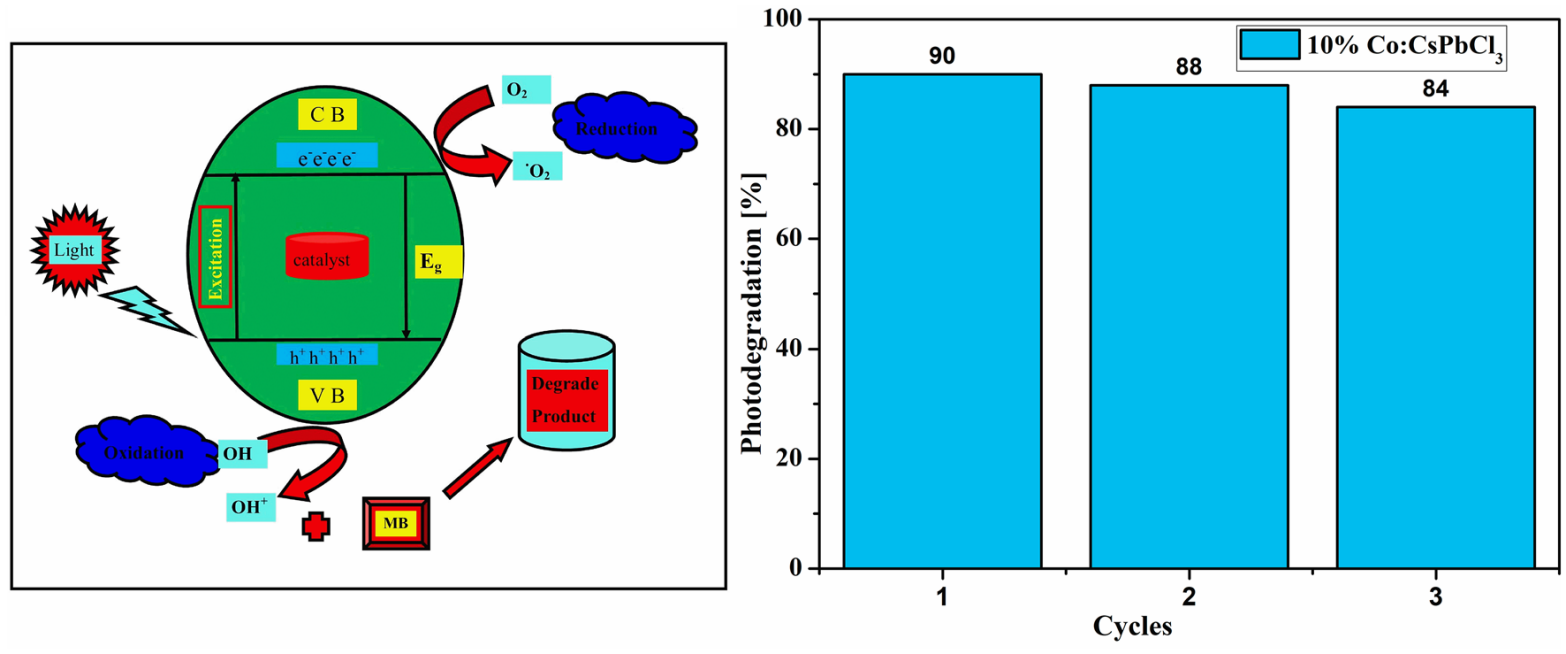
Schematically diagram of photocatlytic process and reusability.

The decoloration observed is believed to have occurred due to photodegradation. If there would have been decoloration (breakage of the chromophores only) the secondary products of the dye would have λ_max_ at some other value (shifted from its usual value) (Fig. 14). Retention of the λ_max_ with simultaneous fall in the absorbance amount confirmed demineralization of the dye molecules^37,38^. The possible mechanism here involved includes first the adsorption of the dye molecules over the catalyst surfaces followed by the adsorption of water molecules. The adsorption here is totally a physical process and is governed by the electrostatic forces between the molecules. When the catalyst treated dye solution was illuminated, the light photons with energy greater than or equal to the band gap of the sample 10% Co:CsPbCl_3_ get absorbed by the catalyst. Absorbed photons result in generation of electrons in the conduction band and holes in the valance band. The generated electron hole pairs get consumed respectively by the adsorbed O_2_ and OH molecules generating highly oxidising free radicals. The hydroxyl free radicals and superoxides thus formed are believed to have oxidised the dye molecules. This mechanism has been illustrated schematically in Fig. 15 and all the steps involved are summerized in equations from Eqs. 6, 7, 8, 9

6 $$ {\text{Co:CsPbCl}}_{3} + {\text{hv}} \to {\text{e}}^{ - } + {\text{h}}^{ + } . $$

7 $$ {\text{O}}_2 + {\text{h}}^{ + } \to^{ \cdot } {\text{O}}_2{ - } $$

8 $$ {\text{H}}_{2} {\text{O}} + {\text{h}}^{ + } \to {\text{OH}}^{ \cdot } + {\text{h}}^{ + } $$

9 $$ {\text{MB}} + {\text{OH}}^{ \cdot } \to {\text{degraded}}\;{\text{dye}} $$

As per definition and industrial value it is very important that the catalyst must have the property of reusability. To check the reusability sample 10% Co:CsPbCl_3_ has been used for three successive cycles and the results are depicted in Fig. 15. For reusability the authors have followed the steps mentioned somewhere in the reported literature^39,40^. During the reusability experiments, the degradation percentage fall by 6% only that showed the potential ability of the nanostructures to act as photocatalysts. The 6% fall might have been resulted due to the possible loss of the catalyst amount while collection after every cycle.

## Conclusion

In summary, we demonistrated the synthesis of CsPbCl_3_ and Co doped CsPbCl_3_ nanostructures by solvothermal method. To the best of knowledge no evedinces of synthesis of Co doped CsPbCl_3_ by solvothermal method are available in the literature. The XRD pattern depicts the formation of CsPbCl_3_ and Co:CsPbCl_3_ in tetragonal phase with space symmetry of P4mm. The thermal stability of synthesised material is estimated by TGA. It is evident from the TG plot that none of the sample shows weight loss up to 600 °C. However there is sharp single-stage degradation of CspbCl_3_ and Co:CsPbCl_3_ without any lower temperature features. Therefore, this material is stable at high temperature as well. SEM micrographs of all the four samples show that the materials are grown in an anisotropic way, which leads to the formation of 3D cubic structures. From the optical studies, it is estimated that these materials are active in the visible region. On increasing the concentration of dopant, the value of absorbance enhances while the band gap reduces from 2.2 to 1.5 eV. The emission spectra were recorded at the excitation wavelength of 360 nm and the emission is recorded nearly at 420 nm. Photoluminescence spectra shows that this material is likely usable in blue LED’s. Finally the electro chemical analysis of CsPbC_3_ and Co:CsPbCl_3_ exhibit the pseudo capacitor behavior. The charge storage capability of synthesized material increases as the concentration of cobalt increases. The 15% Cobalt doped CsPbCl_3_ shows the capacitance of 154 F/g at a scan rate of 10 mV/s making it an essential for storage device applications. Also, the synthesized sample degrades more than 90% of MB dye within 180 min under the illumination of visible light. Hence, from the above discussion, it can be concluded that the Cobalt doping improves all the properties of CsPbCl_3_ mentioned in the present manuscript.
